# *N*-(2-Amino­ethyl)-5-(dimethyl­amino)naphthalene-1-sulfonamide

**DOI:** 10.1107/S160053680901962X

**Published:** 2009-05-29

**Authors:** Shi-lei Zhang, Bi-lin Zhao, Zhen-hong Su, Xian-you Xia, Yong Zhang

**Affiliations:** a School of Chemical and Materials Engineering, Huangshi Institute of Technology, Huangshi 435003, People’s Republic of China; b Medical School, Huangshi Institute of Technology, Huangshi 435003, People’s Republic of China

## Abstract

In the title compound, C_14_H_19_N_3_O_2_S, the N atom of the dimethyl­amino group and the S atom are displaced by 0.078 (2) and 0.084 (2) Å, respectively, from the naphthalene ring plane. The 2-amino­ethyl group has a coiled conformation with an N—C—C—NH_2_ torsion angle of 53.6 (4)°. In the crystal structure, inter­molecular N—H⋯N and weak C—H⋯O hydrogen bonds link mol­ecules into chains along [001].

## Related literature

For applications of ligands containing the 5-(dimethyl­amino)naphthalene-1-sulfonyl (dans­yl) group, see: Corradini *et al.* (1996 ▶, 1997 ▶); Christoforou *et al.* (2006 ▶).
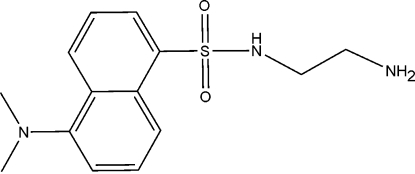


## Experimental

### 

#### Crystal data

C_14_H_19_N_3_O_2_S
*M* *_r_* = 293.38
Orthorhombic, 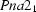

*a* = 15.5221 (15) Å
*b* = 11.5423 (11) Å
*c* = 8.1360 (8) Å
*V* = 1457.7 (2) Å^3^
*Z* = 4
Mo *K*α radiation
μ = 0.23 mm^−1^
*T* = 298 K
0.20 × 0.20 × 0.20 mm

#### Data collection

Bruker SMART CCD diffractometer
Absorption correction: multi-scan (*SADABS*; Sheldrick, 1997 ▶) *T*_min_ = 0.956, *T*_max_ = 0.956
7478 measured reflections
3140 independent reflections
3012 reflections with *I* > 2σ(*I*)
*R*_int_ = 0.029

#### Refinement

*R*[*F* ^2^ > 2σ(*F* ^2^)] = 0.041
*wR*(*F* ^2^) = 0.107
*S* = 1.11
3140 reflections
192 parameters
1 restraint
H atoms treated by a mixture of independent and constrained refinement
Δρ_max_ = 0.24 e Å^−3^
Δρ_min_ = −0.25 e Å^−3^
Absolute structure: Flack (1983 ▶), 1332 Friedel pairs
Flack parameter: −0.03 (8)

### 

Data collection: *SMART* (Bruker, 2007 ▶); cell refinement: *SAINT-Plus* (Bruker, 2007 ▶); data reduction: *SAINT-Plus*; program(s) used to solve structure: *SHELXS97* (Sheldrick, 2008 ▶); program(s) used to refine structure: *SHELXL97* (Sheldrick, 2008 ▶); molecular graphics: *PLATON* (Spek, 2009 ▶); software used to prepare material for publication: *SHELXTL* (Sheldrick, 2008 ▶).

## Supplementary Material

Crystal structure: contains datablocks global, I. DOI: 10.1107/S160053680901962X/lh2821sup1.cif

Structure factors: contains datablocks I. DOI: 10.1107/S160053680901962X/lh2821Isup2.hkl

Additional supplementary materials:  crystallographic information; 3D view; checkCIF report

## Figures and Tables

**Table 1:** Hydrogen-bond geometry (Å, °)

*D*—H⋯*A*	*D*—H	H⋯*A*	*D*⋯*A*	*D*—H⋯*A*
C6—H6⋯O1	0.93	2.48	3.093 (3)	123
N3—H3*A*⋯N2	0.88 (5)	2.52 (6)	2.972 (4)	113 (4)
C11—H11⋯O1^i^	0.93	2.49	3.146 (3)	128
N2—H2*D*⋯N3^ii^	0.87 (3)	2.02 (4)	2.869 (4)	163 (3)

Symmetry codes: (i) 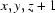; (ii) 
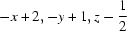
.

**Fig. 1.**
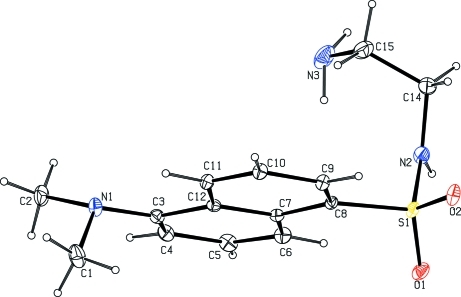
The molecular structure of (I), with displacement ellipsoids drawn at the 50% probability level.

**Fig. 2.**
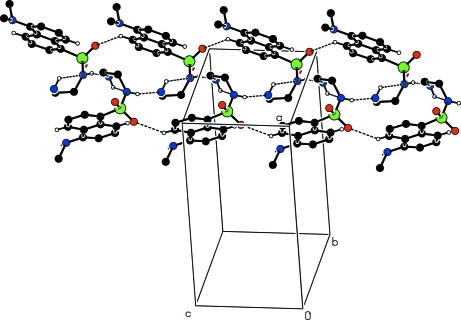
Part of the crystal structure of (I) showing weak hydrogen bonds as dashed lines. Only H atoms involved in hydrogen bonds are shown.

## supplementary crystallographic information

### Comment

The dansyl (5-(dimethylamino)naphthalene-1-sulfonyl) group has been widely used as a fluorophore in the design of fluorescent probes. Recently many fluorescent ligands bearing dansyl group have been reported (Corradini *et al.*, 1996,1997; Christoforou *et al.*, 2006). We are interested in preparing fluorescent ligands that are expected to bind to hydrophobic sites in proteins or membranes. With this mind, the title compound, (I), was prepared and we report the crystal stucture herein.

In the molecule (Fig. 1), atoms N1 and S1 are located approximately in the naphthalene ring plane with their deviations being 0.078 and 0.084 Å, respectively. The  N2—C14—C15—N3 torsion angle of -53.6 (4)° indicates a coiled conformation for the aminoethyl group. In the crystal structure (Fig.2), intermolecular N—H···N and weak C-H···O hydrogen bonds link molecules into one-dimensional chains along [001].

### Experimental

Compound (I) was synthesized according to a literature procedure (Corradini *et al.*, 1996). Single crystals suitable for X-ray diffraction were obtained by slow evaporation of a dichloromethane solution of (I) at room temperature.

### Refinement

All carbon bound H atoms were placed in their idealized positions [*C*—*H*(methyl)=0.96 Å and C—H(aromatic) =0.93 Å] and included in the refinement in the riding-model approximation, with *U*_iso_(methyl H)= 1.5*U*_eq_(C) and *U*_iso_(aromatic H) = 1.2*U*_eq_(C). Hydrogen atoms bonded to nitrogen atoms were found in the difference Fourier maps and refined with the constraints of N—H = 0.869(Å) and *U*_iso_(H) = 1.2*U*_eq_(N).

### Crystal data

**Table:**

C_14_H_19_N_3_O_2_S	*F*(000) = 624
*M**_r_* = 293.38	*D*_x_ = 1.337 Mg m^−^^3^
Orthorhombic, *P**n**a*2_1_	Mo *K*α radiation, λ = 0.71073 Å
Hall symbol: P 2c -2n	Cell parameters from 4085 reflections
*a* = 15.5221 (15) Å	θ = 2.2–28.0°
*b* = 11.5423 (11) Å	µ = 0.23 mm^−^^1^
*c* = 8.1360 (8) Å	*T* = 298 K
*V* = 1457.7 (2) Å^3^	Block, colorless
*Z* = 4	0.20 × 0.20 × 0.20 mm

### Data collection

**Table:**

Bruker SMART CCD diffractometer	3140 independent reflections
Radiation source: fine-focus sealed tube	3012 reflections with *I* > 2σ(*I*)
graphite	*R*_int_ = 0.029
φ and ω scans	θ_max_ = 27.0°, θ_min_ = 2.2°
Absorption correction: multi-scan (*SADABS*; Sheldrick, 1997)	*h* = −19→13
*T*_min_ = 0.956, *T*_max_ = 0.956	*k* = −14→14
7478 measured reflections	*l* = −10→10

### Refinement

**Table:**

Refinement on *F*^2^	Secondary atom site location: difference Fourier map
Least-squares matrix: full	Hydrogen site location: inferred from neighbouring sites
*R*[*F*^2^ > 2σ(*F*^2^)] = 0.041	H atoms treated by a mixture of independent and constrained refinement
*wR*(*F*^2^) = 0.107	*w* = 1/[σ^2^(*F*_o_^2^) + (0.0621*P*)^2^ + 0.0989*P*] where *P* = (*F*_o_^2^ + 2*F*_c_^2^)/3
*S* = 1.11	(Δ/σ)_max_ < 0.001
3140 reflections	Δρ_max_ = 0.24 e Å^−^^3^
192 parameters	Δρ_min_ = −0.25 e Å^−^^3^
1 restraint	Absolute structure: Flack (1983), 1332 Friedel pairs
Primary atom site location: structure-invariant direct methods	Flack parameter: −0.03 (8)

### Special details

**Table:**

Geometry. All e.s.d.'s (except the e.s.d. in the dihedral angle between two l.s. planes) are estimated using the full covariance matrix. The cell e.s.d.'s are taken into account individually in the estimation of e.s.d.'s in distances, angles and torsion angles; correlations between e.s.d.'s in cell parameters are only used when they are defined by crystal symmetry. An approximate (isotropic) treatment of cell e.s.d.'s is used for estimating e.s.d.'s involving l.s. planes.
Refinement. Refinement of *F*^2^ against ALL reflections. The weighted *R*-factor *wR* and goodness of fit *S* are based on *F*^2^, conventional *R*-factors *R* are based on *F*, with *F* set to zero for negative *F*^2^. The threshold expression of *F*^2^ > σ(*F*^2^) is used only for calculating *R*-factors(gt) *etc*. and is not relevant to the choice of reflections for refinement. *R*-factors based on *F*^2^ are statistically about twice as large as those based on *F*, and *R*- factors based on ALL data will be even larger.

### Fractional atomic coordinates and isotropic or equivalent isotropic displacement parameters (Å^2^)

**Table:**

	*x*	*y*	*z*	*U*_iso_*/*U*_eq_	
C1	0.6783 (2)	0.2592 (3)	1.2600 (5)	0.0711 (9)	
H1A	0.6195	0.2620	1.2236	0.107*	
H1B	0.6797	0.2519	1.3775	0.107*	
H1C	0.7065	0.1937	1.2111	0.107*	
C2	0.67195 (17)	0.4676 (3)	1.2579 (4)	0.0615 (7)	
H2A	0.7030	0.5368	1.2300	0.092*	
H2B	0.6616	0.4659	1.3742	0.092*	
H2C	0.6180	0.4665	1.2004	0.092*	
C3	0.75553 (12)	0.36601 (19)	1.0498 (3)	0.0381 (5)	
C4	0.72410 (14)	0.43583 (19)	0.9255 (3)	0.0451 (5)	
H4	0.6796	0.4874	0.9477	0.054*	
C5	0.75833 (15)	0.4299 (2)	0.7670 (3)	0.0480 (6)	
H5	0.7350	0.4767	0.6853	0.058*	
C6	0.82454 (15)	0.3580 (2)	0.7281 (3)	0.0445 (5)	
H6	0.8451	0.3547	0.6208	0.053*	
C7	0.86251 (12)	0.28748 (17)	0.8530 (3)	0.0323 (4)	
C8	0.93538 (13)	0.21356 (16)	0.8264 (3)	0.0309 (4)	
C9	0.96977 (14)	0.14947 (18)	0.9519 (3)	0.0366 (4)	
H9	1.0158	0.0999	0.9311	0.044*	
C10	0.93643 (14)	0.15783 (18)	1.1106 (3)	0.0379 (4)	
H10	0.9610	0.1151	1.1954	0.045*	
C11	0.86816 (12)	0.22823 (16)	1.1418 (3)	0.0363 (4)	
H11	0.8473	0.2345	1.2486	0.044*	
C12	0.82814 (12)	0.29230 (16)	1.0143 (3)	0.0322 (4)	
C14	1.10343 (19)	0.3731 (3)	0.6827 (4)	0.0689 (9)	
H14A	1.1354	0.4225	0.6080	0.083*	
H14B	1.1400	0.3079	0.7107	0.083*	
C15	1.0839 (2)	0.4406 (3)	0.8375 (5)	0.0833 (11)	
H15A	1.0609	0.3874	0.9188	0.100*	
H15B	1.1375	0.4712	0.8805	0.100*	
N1	0.72278 (13)	0.36630 (17)	1.2108 (3)	0.0467 (5)	
O1	0.92413 (13)	0.18510 (17)	0.5084 (2)	0.0558 (5)	
O2	1.05529 (12)	0.12231 (15)	0.6510 (2)	0.0553 (4)	
N2	1.02722 (14)	0.33007 (18)	0.5987 (3)	0.0495 (5)	
H2D	1.0054 (19)	0.358 (3)	0.508 (5)	0.059*	
N3	1.0237 (3)	0.5358 (3)	0.8182 (5)	0.0993 (13)	
H3A	0.980 (3)	0.499 (5)	0.773 (8)	0.119*	
H3B	1.044 (3)	0.588 (4)	0.752 (7)	0.119*	
S1	0.98740 (3)	0.20448 (4)	0.63277 (8)	0.03840 (15)	

### Atomic displacement parameters (Å^2^)

**Table:**

	*U*^11^	*U*^22^	*U*^33^	*U*^12^	*U*^13^	*U*^23^
C1	0.0693 (17)	0.0655 (18)	0.079 (2)	0.0010 (15)	0.0311 (15)	0.0156 (17)
C2	0.0547 (14)	0.0663 (17)	0.0635 (18)	0.0210 (13)	0.0108 (12)	−0.0083 (14)
C3	0.0340 (10)	0.0365 (11)	0.0439 (12)	0.0001 (8)	−0.0005 (9)	−0.0006 (9)
C4	0.0377 (10)	0.0406 (12)	0.0570 (14)	0.0071 (9)	−0.0008 (10)	0.0082 (11)
C5	0.0472 (12)	0.0486 (13)	0.0482 (14)	0.0062 (10)	−0.0097 (10)	0.0171 (11)
C6	0.0533 (12)	0.0461 (12)	0.0343 (11)	−0.0003 (10)	−0.0053 (9)	0.0120 (10)
C7	0.0331 (9)	0.0315 (9)	0.0323 (10)	−0.0037 (7)	−0.0041 (8)	0.0023 (8)
C8	0.0369 (10)	0.0294 (9)	0.0264 (10)	−0.0018 (8)	0.0014 (8)	−0.0008 (8)
C9	0.0418 (10)	0.0344 (11)	0.0336 (10)	0.0061 (8)	−0.0015 (9)	0.0015 (8)
C10	0.0450 (10)	0.0387 (10)	0.0299 (11)	0.0066 (8)	−0.0045 (8)	0.0077 (8)
C11	0.0434 (10)	0.0367 (9)	0.0286 (9)	0.0018 (7)	0.0003 (9)	0.0037 (9)
C12	0.0328 (9)	0.0303 (10)	0.0335 (10)	−0.0049 (7)	−0.0015 (8)	0.0030 (8)
C14	0.0647 (16)	0.0609 (16)	0.081 (3)	−0.0208 (14)	0.0173 (15)	−0.0189 (15)
C15	0.099 (2)	0.077 (2)	0.075 (2)	−0.034 (2)	0.012 (2)	−0.0264 (19)
N1	0.0462 (10)	0.0458 (11)	0.0482 (11)	0.0089 (9)	0.0133 (9)	0.0015 (9)
O1	0.0778 (12)	0.0595 (11)	0.0302 (8)	−0.0089 (9)	−0.0003 (8)	−0.0045 (8)
O2	0.0729 (10)	0.0498 (9)	0.0432 (9)	0.0162 (8)	0.0174 (9)	−0.0028 (8)
N2	0.0641 (13)	0.0425 (10)	0.0420 (14)	−0.0092 (9)	0.0122 (9)	0.0037 (9)
N3	0.158 (4)	0.0527 (18)	0.087 (2)	−0.0261 (19)	0.036 (2)	−0.0248 (16)
S1	0.0538 (3)	0.0341 (2)	0.0274 (2)	−0.00129 (19)	0.0066 (3)	−0.0034 (3)

### Geometric parameters (Å, °)

**Table:**

C1—N1	1.472 (3)	C8—S1	1.773 (2)
C1—H1A	0.9600	C9—C10	1.395 (3)
C1—H1B	0.9600	C9—H9	0.9300
C1—H1C	0.9600	C10—C11	1.359 (3)
C2—N1	1.461 (3)	C10—H10	0.9300
C2—H2A	0.9600	C11—C12	1.417 (3)
C2—H2B	0.9600	C11—H11	0.9300
C2—H2C	0.9600	C14—N2	1.454 (4)
C3—C4	1.382 (3)	C14—C15	1.511 (5)
C3—N1	1.405 (3)	C14—H14A	0.9700
C3—C12	1.441 (3)	C14—H14B	0.9700
C4—C5	1.396 (3)	C15—N3	1.450 (5)
C4—H4	0.9300	C15—H15A	0.9700
C5—C6	1.358 (3)	C15—H15B	0.9700
C5—H5	0.9300	O1—S1	1.4278 (19)
C6—C7	1.429 (3)	O2—S1	1.4255 (18)
C6—H6	0.9300	N2—S1	1.600 (2)
C7—C12	1.417 (3)	N2—H2D	0.87 (3)
C7—C8	1.433 (3)	N3—H3A	0.88 (5)
C8—C9	1.369 (3)	N3—H3B	0.87 (5)
			
N1—C1—H1A	109.5	C9—C10—H10	119.9
N1—C1—H1B	109.5	C10—C11—C12	121.1 (2)
H1A—C1—H1B	109.5	C10—C11—H11	119.4
N1—C1—H1C	109.5	C12—C11—H11	119.4
H1A—C1—H1C	109.5	C11—C12—C7	119.47 (18)
H1B—C1—H1C	109.5	C11—C12—C3	120.3 (2)
N1—C2—H2A	109.5	C7—C12—C3	120.20 (18)
N1—C2—H2B	109.5	N2—C14—C15	113.9 (3)
H2A—C2—H2B	109.5	N2—C14—H14A	108.8
N1—C2—H2C	109.5	C15—C14—H14A	108.8
H2A—C2—H2C	109.5	N2—C14—H14B	108.8
H2B—C2—H2C	109.5	C15—C14—H14B	108.8
C4—C3—N1	123.6 (2)	H14A—C14—H14B	107.7
C4—C3—C12	118.3 (2)	N3—C15—C14	115.5 (3)
N1—C3—C12	118.11 (19)	N3—C15—H15A	108.4
C3—C4—C5	120.8 (2)	C14—C15—H15A	108.4
C3—C4—H4	119.6	N3—C15—H15B	108.4
C5—C4—H4	119.6	C14—C15—H15B	108.4
C6—C5—C4	122.3 (2)	H15A—C15—H15B	107.5
C6—C5—H5	118.9	C3—N1—C2	116.2 (2)
C4—C5—H5	118.9	C3—N1—C1	114.9 (2)
C5—C6—C7	119.6 (2)	C2—N1—C1	110.3 (2)
C5—C6—H6	120.2	C14—N2—S1	122.8 (2)
C7—C6—H6	120.2	C14—N2—H2D	126 (2)
C12—C7—C6	118.73 (19)	S1—N2—H2D	109 (2)
C12—C7—C8	117.42 (17)	C15—N3—H3A	100 (3)
C6—C7—C8	123.8 (2)	C15—N3—H3B	111 (3)
C9—C8—C7	121.08 (19)	H3A—N3—H3B	111 (5)
C9—C8—S1	116.94 (16)	O2—S1—O1	118.56 (12)
C7—C8—S1	121.94 (15)	O2—S1—N2	109.58 (12)
C8—C9—C10	120.57 (19)	O1—S1—N2	106.55 (12)
C8—C9—H9	119.7	O2—S1—C8	106.46 (10)
C10—C9—H9	119.7	O1—S1—C8	109.01 (10)
C11—C10—C9	120.2 (2)	N2—S1—C8	106.04 (10)
C11—C10—H10	119.9		
			
N1—C3—C4—C5	−178.7 (2)	C4—C3—C12—C11	173.49 (19)
C12—C3—C4—C5	3.9 (3)	N1—C3—C12—C11	−4.0 (3)
C3—C4—C5—C6	−1.4 (4)	C4—C3—C12—C7	−3.6 (3)
C4—C5—C6—C7	−1.5 (4)	N1—C3—C12—C7	178.89 (18)
C5—C6—C7—C12	1.7 (3)	N2—C14—C15—N3	−53.6 (4)
C5—C6—C7—C8	−176.60 (19)	C4—C3—N1—C2	−18.4 (3)
C12—C7—C8—C9	0.6 (3)	C12—C3—N1—C2	159.0 (2)
C6—C7—C8—C9	178.9 (2)	C4—C3—N1—C1	112.6 (3)
C12—C7—C8—S1	−177.20 (14)	C12—C3—N1—C1	−70.0 (3)
C6—C7—C8—S1	1.1 (3)	C15—C14—N2—S1	−91.9 (3)
C7—C8—C9—C10	−2.3 (3)	C14—N2—S1—O2	−40.1 (2)
S1—C8—C9—C10	175.56 (16)	C14—N2—S1—O1	−169.58 (19)
C8—C9—C10—C11	1.3 (3)	C14—N2—S1—C8	74.4 (2)
C9—C10—C11—C12	1.5 (3)	C9—C8—S1—O2	3.06 (19)
C10—C11—C12—C7	−3.2 (3)	C7—C8—S1—O2	−179.09 (16)
C10—C11—C12—C3	179.68 (19)	C9—C8—S1—O1	132.05 (17)
C6—C7—C12—C11	−176.28 (19)	C7—C8—S1—O1	−50.11 (19)
C8—C7—C12—C11	2.1 (3)	C9—C8—S1—N2	−113.59 (18)
C6—C7—C12—C3	0.8 (3)	C7—C8—S1—N2	64.25 (19)
C8—C7—C12—C3	179.24 (17)		

### Hydrogen-bond geometry (Å, °)

**Table:**

*D*—H···*A*	*D*—H	H···*A*	*D*···*A*	*D*—H···*A*
C6—H6···O1	0.93	2.48	3.093 (3)	123
N3—H3A···N2	0.88 (5)	2.52 (6)	2.972 (4)	113 (4)
C11—H11···O1^i^	0.93	2.49	3.146 (3)	128
N2—H2D···N3^ii^	0.87 (3)	2.02 (4)	2.869 (4)	163 (3)

Symmetry codes: (i) *x*, *y*, *z*+1; (ii) −*x*+2, −*y*+1, *z*−1/2.
